# Sustainable graphitic carbon from biomass to be suitable for technological devices

**DOI:** 10.55730/1300-0527.3621

**Published:** 2023-10-16

**Authors:** Gökçen AKGÜL

**Affiliations:** Department of Energy Systems Engineering, Engineering and Architecture Faculty, Recep Tayyip Erdoğan University, Rize, Turkiye

**Keywords:** Biomass, graphitic nanocarbon, electrical conductive carbonaceous material

## Abstract

Technological devices are mostly manufactured by conductive and semiconductive materials. As advancement in the last decades, carbon nanomaterials have been explored in electrical/electronic technology due to their unique performances for manufacturing developing, and prudential miniaturized and flexible electrical/electronic devices. In the era of sustainable and clean carbon technology; renewable, alternative, biodegradable, and eco-friendly new carbon resources are required. Biomass could be the answer to offer inspiring carbon allotropes from nature to be suitable for developing electrical/electronic devices. In this article, deriving of the technological carbonaceous material from biomass, studies although they are very limited in the literature on obtaining the electrical conductive ones and the progress as electrical conductive renewable material are presented.

## 1. Introduction

Technological devices, namely the electrical and electronic ones, are mostly manufactured by conductive (Cu, Ag, Au, Pt, etc.) and semiconductive materials (Si, Ge, Ga, etc.) and the alloys of groups of III-V and II–VI since 1950 [1]. Beginning in the 1980s, carbon-based high molecular weight polymer materials were added into the conductive/semiconductive materials’ inbox with the advantages of flexibility, encapsulation, special phases (liquid crystals e.g.,) and optical features. However, their usages were restricted because of their temperature-chemical instability or statistical/electrical polarizations [2]. As advancement in the last decades, carbon nanomaterials have been explored in electrical/electronic technology due to their unique high performances such as electrical conductivity, charge mobility, thermal stability, and flexibility [3]. By reduced dimensions offering superior optical, mechanical, and thermal properties like high electron mobility, high charge carrying, high crystallinity, adjustable energy band gap, enhanced electrostatics etc. compared to the metallic bulk ones, carbon nanomaterials have opened a new window for manufacturing the developing and prudential miniaturized and flexible electrical/electronic devices such as foldable TV or cell phone displaces, tiny chips, wearable gadgets, printable solar cells, antennas, photovoltaics or sensors to various revolutionary smart devices [4–6].

Carbon has various allotropes with different hybridization, chirality, and orientation of the atoms that are diversifying the electric/electronic applications. The allotropes can be classified into 3 general groups; (i) diamond (and like) structure tetrahedral configured with sp^3^ bonded carbon, (ii) graphite and derived materials such as graphene, fullerene, carbon nanotube (bamboo like cup-stacked, nano horns, helical carbon nanotube (CNT), etc.), quantum dots, carbon onion or some other hexagonal configurated structures with sp^2^ hybridization, and (iii) sp hybridized structures refer to carbine or like materials. Other predicted or experimentally synthesized carbon forms with mixing hybridization also exist as transitional allotropes such as schwartizes, fullerite, graphyne, graphdiyne and amorphous carbon [7,8]. The ternary phase diagram summarizes the classification based on the hybridization [9].

**Figure 1:**
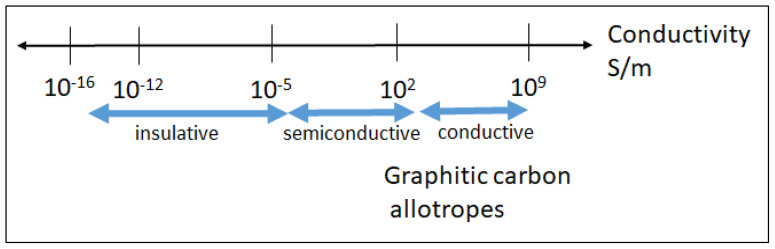
Conductivity levels

The enhanced electron delocalization on the sp^2^ bonds and quantum confinement of electrons are mostly responsible for the superior electrical conductivity of the carbon nanomaterials that is the decisive factor for the potential electrical/electronic applications. Therefore, particularly the graphitic carbon allotropes (graphene, CNT, carbon nanohybrids like nano onions, etc.) with more sp^2^ hybridization have attracted interest in electronic-based industries as alternatives to silicon technology (Figure 1). CNT, for instance, is recommended as Moore’s Law materials to chip manufacturing enterprises by the organisation of the International Roadmap for Devices and Systems [10]. CNT has also been evaluated as a replacement material for conductive wire by Cu or Al for long distance metal free electricity transfer [11]. Graphene and CNT-based high sensible sensors and detectors, optoelectronics and photonics (e.g., long distance communication devices), field effect transistors, logic/transformable/integrated circuits, electromechanical capacitors are already some implementations with superior electrical properties [3,12–15].

Besides the exciting properties of graphitic allotropes in electrical/electronic implementations mentioned above [3–5], the challenges are mass production and technical troubles in quality by the synthesis. Commercially, carbon nanomaterials are mostly produced from fossil sources; graphite from coal [16], fullerenes and CNT from hydrocarbons [17] and graphene by utilizing methane gas [18] are some examples. In the era of sustainable and clean carbon technology in electric/electronics; renewable, alternative, biodegradable, and eco-friendly new carbon resources are required.

Biomass could be the answer to offer inspiring carbon allotropes from nature. While the digital, electronic and electrical world is ever growing in the most part of miniaturization with the help of carbon nanomaterials, modern and flexible electrical/electronic devices could be achieved by utilization of biomass as a renewable and sustainable carbon resource since the formation of the biomass resources does not take millions of years like fossil sources.

In this article, deriving graphitic carbon from biomass to be suitable for developing electrical/electronic devices is mini-reviewed. In the following sections, the general routes from pristine biomass to refined carbon, studies on obtaining electrical conductive carbonaceous materials although there are very limited in the literature and its progress as electrical conductive renewable material are presented.

## 2. Graphitic/electrical conductive carbon from biomass

Carbon is the skeleton element of organic molecules especially in living organisms. “The mass of living organisms, including plants, animals, and microorganisms, or, from a biochemical perspective, cellulose, lignin, sugars, fats, and proteins” are called biomass by Houghton [19] in the Encyclopedia of Ecology. *“Biomass includes both the above- and below-ground tissues of plants, for example, leaves, twigs, branches, boles, as well as roots of trees and rhizomes of grasses”* that it is the only renewable carbon resource.

**Figure 2:**
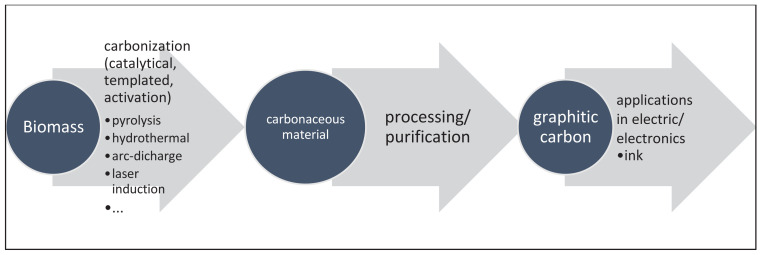
The general pathway towards graphitic carbon from biomass

In the last decades, naturally available biomass has been gaining attention from researchers and manufacturers on the derivation and production of electrical conductive graphitic carbon. Xu et al. [20] reviewed the biomass valorization to carbon nanomaterials in the “waste to wealth” concept. The diverse biomass resources like fish or shrimp waste, animal manures, lignocellulosic materials, domestic and sewage wastes, rice husks, etc. are converted by their distinctive processes to value-added products; such as fullerenes, graphitic/graphenic carbons [21], CNT and nanodiamonds. The general pathway towards graphitic carbon from biomass is given in Figure 2. The first step towards electrical conductive graphitic carbon is to carbonize the biomass resources which can be performed by various thermal treatments such as pyrolysis, hydrothermal carbonization, arc-discharge heating, molten salt templated carbonization, plasma assisted methods or Joule heating [22,23]. Catalysts like salts or particles of metals are commonly used to promote the graphitization during the carbonization processes [24–27]. Partially inert or fully inert atmospheres supplied by argon or nitrogen gases are ensured in the processes to obtain carbonaceous material instead of combusted one.

**Figure 3:**
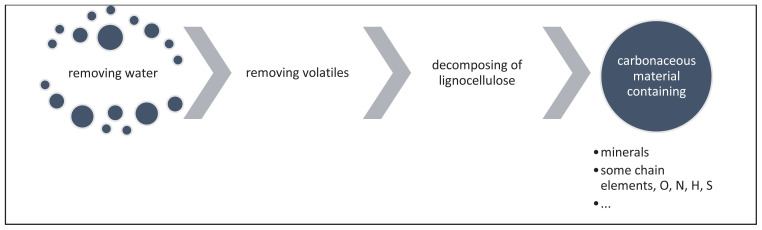
The general pathway to fixed carbon from biomass by heat treatment

The carbonaceous material is eventually not pure carbon or graphitic carbon. Since the pristine biomass consists of various ingredients such as molecules of cellulose-hemicellulose-lignin, fat, vitamins, or proteins, carbonaceous material would include structural elements other than carbon such as oxygen, nitrogen, sulphur, phosphorus, hydrogen; and some minerals like potassium, sodium, calcium, etc. During the carbonization processes, the humidity and the volatile matter (carbon monoxide, light acids (formic acid, acetic acid) methanol, and some furfurals…) [28] are removed from biomass first, following structural ordering by reducing oxygen content (Figure 3) to more graphitic structures [29] and the end product would still be a mixture of graphitic and amorphous carbon with structural elements and minerals [30].

**Figure 4:**
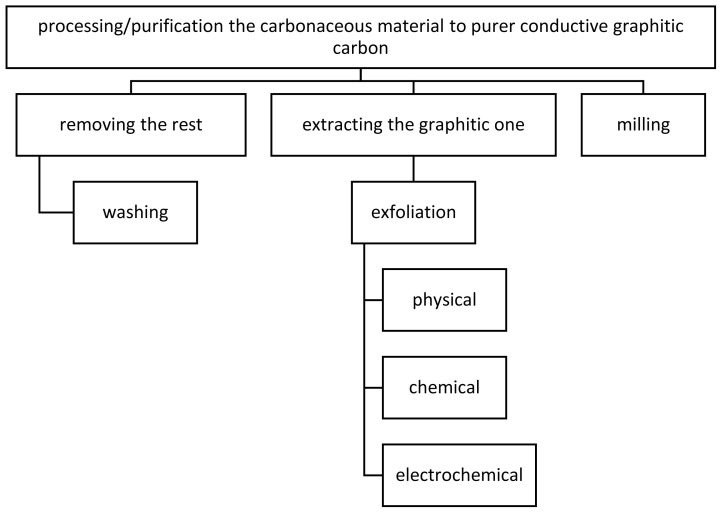
Processing the carbonaceous mixture

The mixture of carbonaceous material is processed by two main routes towards purer conductive graphitic carbon by; (i) removing the rest from the mixture or (ii) extracting the graphitic part from the mixture (Figure 4). Milling or grinding would also help to maintain the nano-sized graphitic carbons.

As the processing method, removing the rest apart from the graphitic part from the mixture could be performed by washing or dispersing the carbonaceous material. Acid washing would efficiently pull out the minerals [31]. Hoffman et al. [32] explained a lower electrical conductivity of the raw pruning vineyard biochar due to high mineral/ash content.

Extracting the graphitized carbon inside the carbonized biomass sample could directly be performed by physical, chemical, or electrochemical exfoliation methods which are especially beneficial to extract the graphene and graphene-like carbons [33].

The scotch tape method is the classical physical method but it is not suitable for large scale production. On the other hand, dispersing carbonized biomass in a solution (mostly in water) would help to extract the nanoparticles from the mixture physically. Dong et al. [34] obtained nano-sized conductive biochar attained from bagasse by pyrolysis. The carbonaceous material was suspended in water first, then centrifuged so that the supernatant containing nano-sized biochar particles was collected for preparation of a real environmental water sensor.

Modified Hummers’ method with assisting sonication is the popular chemical exfoliation method to extract graphenic structures from graphitic ones. Lu et al. [35] obtained graphene-like conductive carbon from fresh lotus biomass by chemical exfoliation. The biomass structure was drilled with oxidation by peroxide and hydrolyzed by acetic acid resulting in graphene-like carbon with a conductivity of 1.23 S.

The electrochemical exfoliation has various advantages for mass, high quality and low-cost production of graphene from the sources. This method implies that electrical current is realized between 2 or 3 electrodes (at least 1 is the carbon precursor, typically rod or foil carbon electrode) immersed in an electrolyte of which ions intercalate among the graphene layers and cause expansion and exfoliation of graphene structures through various reactions [36]. This “wet” chemical dispersion is mostly assisted by sonochemical methods which refers to the process of utilizing sound energy to discrete the layered particles. There would be found profusely studies in the literature on the electrochemical exfoliation of graphite to graphene, however, studies on the exfoliation of graphitized carbon from biomass to purer graphitic or graphenic structures are still in deficiency although some flush or high heat treatments of biomass are also called as exfoliation [37].

As the third main practice, the grinding process could also be an effective method for the development of nanoparticles, consequently, in the development of conductive carbon materials from biomass. Lyu et al. [38] reported that reduction the particle size of the carbonaceous material (biochar) derived from bamboo and sugarcane bagasse by ball milling increases the electrical conductivity of the carbonaceous material around by ten fold from 0.1 S/m to 1 S/m as a result of enhanced surface area, internal nano pore networks and enhanced electron exchange capability between the electrode and the electrolyte by nano pores.

Although it may seem very troublesome at first glance to obtain graphitic carbon from biomass as a result of so many processes, however, all these processing/purification processes even including carbonization are already applied to obtain commercial graphitic or graphenic materials from fossil fuels [16–18]. Therefore, biomass maintains its place as a clean, renewable, sustainable, and alternative conductive carbon resource.

## 3. Studies in the literature

Acquiring biomass-based carbon nanomaterials is among the current research topics and very open to improvements. Recently, Tiwari et al. [23] and Tamuly et al. [21] reviewed the conversion of biomass into value-added graphene and graphene-like porous carbon nanomaterials and their applications. They stated the advantages of the transformation of biomass as a low-cost and widely available resource and a wide range of applications of carbon nanostructures derived from biomass from energy storage, sensing devices, or waste water treatment to CO_2_ adsorption.

Jiang et al. [39] demonstrated the conversion of lignin into highly ordered graphitic and electrical conductive carbon with the value of 640 S/m obtained by only carbonization with Joule heating process at ultrahigh temperatures. Further, when a templated method was applied in the study, the electrical conductivity could be enhanced to 4.5·10^5^ S/m as high as graphite by highly ordering the crystalline structure of carbonaceous material from lignin.

The formation of nano-layered graphitic structures by pyrolysis of bamboo and pine biomass at 600–1300 °C was investigated by Gezahegn et al. [37]. The highest electrical conductivity is determined for the bamboo biochar as 1.9·10^5^ S/m produced by pyrolysis at 1300 °C, 2h residence time and 4.7 °C/min heating rate. Increasing the residence time and temperature at a lower heating rate has resulted in the increase in graphitic and single crystal type structures.

Hoffmann et al. [32] compared the electrical conductivities of carbon samples generated from vineyard and cellulose biomass resources by pyrolysis, hydrothermal treatment, and both carbonization methods. While the cellulose-derived carbon by hydrothermal treatment has almost any conductivity, it increases to 51 S/m with the process of pyrolysis at 900 °C for 2h. Application of both processes one after another improves the electrical conductivity to 179 S/m. On the other hand, vineyard biomass (pruning) originated carbon has shown lower conductivity at the same experimental conditions explained as the result of different conversion patterns of lignocellulosic biomass, less π systems, less aromatization, and volatilization. However, it was predicted that the carbon properties could be tailored to desired conductive products by various carbonization methods.

Since the carbonized biomass would still have an amorphous structure, it can be graphitized by further treatments such as electrochemical treatments. Jin et al. [40] presented that graphitization of amorphous carbon to nano-structured graphites can be converted by electrochemical methods in template molten salt at 1100 K and inert atmosphere of Ar.

Activation, increasing the surface area and scraping the particles to nano sizes by chemicals, is another treatment performed during the carbonization of biomass to obtain a better electrical conductive carbon material. Celiktas and Alptekin [41] presented that hazelnut shell, nut shell and corn cob biomass derived carbonaceous materials’ conductivities can be improved by activation with the aid of developing the porosity and particle sizes. The biomass resources were carbonized at hydrothermal conditions at 250 °C, then activated by various chemicals of NaOH, KOH, ZnCl_2,_ and H_3_PO_4_ and pyrolyzed at 800 °C. They found out that NaOH-activated carbon material has higher electrical conductivity than nonactivated ones.

Gabhi et al. [42] presented that the electrical conductivity of biomass-derived carbon is highly sensitive to carbonization/graphitization degree by increasing the sp^2^ electron configuration and lowering the O/C and H/C atomic ratio. It is indicated that while the sugar maple wood biomass is an insulator, the electrical conductivity of the derived biochar sample with a higher carbonization degree of more than 85% and higher graphitization can reach to 400 S/m which is higher than graphite single crystal structure in a direction perpendicular to graphene plane electrical conductivity (333.3 S/m).

Further, some researchers are defending the noncarbonized, piristine, and well-crystallized biomass utilizing electronics as conductive material with the help of doping properties and facile modification of biomass than carbonized ones [43]. The Table summarizes some studies on the graphitic carbon derivation and their conductivities.

The results of the huge conductivity differences given in the Table would be reasoned of the factors; (i) carbon derivation methods (pyrolysis, hydrothermal or sequential processes, etc.) and experimental parameters even at the same procedure which affect the characteristics of the carbon material very much, (ii) conductivity measurement methods. The electrical conductivities of the carbon samples can be determined by various methods; electrochemical impedance spectroscopy (EIS), guard ring/compressive strain, 4-point methods, or basically with a multimeter of the pressed samples. The main differences among these methods are the applied potential or current type as alternating (AC), direct (DC) ones, or magnetic field applications. In the common ground, the volumetric or surface resistivity is determined by all methods. While EIS measures the generally volumetric resistivity (impedance) by AC, the guard ring method consists of two pistons and a guard electrode that the volumetric or surface contact resistance between the electrode and carbon powder is determined under certain pressure by DC. The 4-point methods are utilized for thin film samples where the surface resistivities are determined in a magnetic field by DC. The easiest and rough resistance measurement of a material is made by a multimeter by injecting a small current and determining the total resistance including cable, substrate, sample, and contacts.

Eventually, pyrolysis or catalytic pyrolysis is the common graphitization method for the derivation of graphitic carbon from biomass with the heat application at 600–1300 °C in the presence of a catalyst or template at the inert atmosphere provided by N_2_ or Ar [50,51].

The final conductive carbonaceous product from biomass can be realized as ink relatively easily to produce electronic devices [52,53]. Printing technology for the manufacturing and utilization of electrical/electronic devices is beneficial in terms of patterning the devices due to selective deposition, minimization of energy consumption, low cost, and saving the materials. Mass manufacturing of thin, bendable, lightweight, and elastic/flexible electronics is available by printing technology. Additionally, it is an environmentally friendly method that does not need hazardous chemicals for etching.

Poulin et al. [54] prepared a conductive ink composed of carbon particles (graphite and carbon black) dispersed in a solution of shellac which is a kind of organic resin used mostly by furniture manufacturers as polish material. They demonstrated that this ecologically friendly ink is compatible and relevant for disposable, short-lived printed electronic devices. Claypole et al. [55] compared the electrical conductivity and stretchability performance of the carbon ink prepared by ammonia plasma functionalized graphite nano platelets-carbon black in thermoplastic polyurethane/diacetone alcohol solvent with silver ink in the same polymer/solvent system. The resistance of the silver ink is considerably lower than the carbon ink but carbon ink showed consistent electrical response to applied strain while the resistance of the silver ink increased. Consequently, highly electrical conductive inks were improved by combining the carbon nanomaterials with the silver nanoparticle fillers to enhance the electrical performance by metallic bulk conductivities.

Commercial conductive carbon inks have various ingredients for smooth printing assembled the carbon nanomaterials with a suitable solvent, stabilizer, and binder. The solvents provide a better dispersion of the mixture while the stabilizer, mostly a proper polymer material is utilized, prevents redispersion of nanosized carbon particles in air. The other ingredient binders improve the interactions between carbon-carbon and carbon-substrate so that stability and stretchability on the substrate can be achieved. The ink should provide at least 100 S/m electrical conductivity [54].

The studies on conductive carbon derivation from renewable carbon resources of biomass are still at the beginning stage, however, “Dual Carbon” called biomass-derived carbons are already concerned in energy storage batteries [56].

## 4. Conclusion

All products in the future need to be smart, low energy consumer, and eco-friendly at the same time. Carbon nanomaterials offer superior optical, mechanical, and thermal properties for manufacturing developing and prudential miniaturized, flexible, and smart electrical/electronic devices. In this work, the importance of the nanocarbon materials and their derivation potential from renewable, sustainable, and clean carbon resources of biomass and their concern as electrical conductive carbonaceous material in electrical/electronic technology is summarized. Very little is known about renewable and technological carbonaceous materials from biomass and more research and visualizations are needed. This subject is highly interesting to open a gate for more sustainable and renewable developments by using natural sources.

## Figures and Tables

**Table:** Electrical conductivities of carbonaceous materials derived from biomass.

Biomass	Procedure	Conductivity	Conductivity Measurement method	Reference
Lotus	Hydrothermal treatment and pyrolysis	1.23 S	EIS	[34]
Cabbage leaves	Hydrothermal treatment, freeze-drying, and pyrolysis	1.47 S	EIS	[44]
Poplar catkin	Activating, pyrolysis	47 S/m	Compressive strain	[45]
Vineyard residue (pruning)	Hydrothermal carbonization, pyrolysis	100 S/m	Compressive strain	[32]
Cellulose	Hydrothermal carbonization, pyrolysis	179 S/m	Compressive strain	[32]
Cellulose nanofiber	Femtosecond laser induced graphitization	690 S/m	2 probe method	[46]
Cow dung	Pyrolysis	6.84·10^6^ S/m	4 probe method	[47]
Bamboo	Pyrolysis	1.9 ·10^5^ S/m bamboo	4 probe analyzer	[36]
Bamboo	Pyrolysis	236 S/m	2-probe bed technique with compressing	[48]
Lignin	GO-lignin film, carbonization, Joule heating	4.5 ·10^5^ S/m	4 probe analyzer	[39]
Chitin nanofiber paper	Pyrolysis	10^4^ S/m	Resistivity meter	[49]
